# Numerical Methods and Applications in Biomechanical Modeling

**DOI:** 10.1155/2013/727830

**Published:** 2013-11-26

**Authors:** Eddie Y. K. Ng, Harvey S. Borovetz, Eduardo Soudah, Zhonghua Sun

**Affiliations:** ^1^Nanyang Technological University, School of Mechanical and Aerospace Engineering, Singapore 639798; ^2^Department of Bioengineering, University of Pittsburgh, Pittsburgh, PA 15261, USA; ^3^International Center for Numerical Methods in Engineering (CIMNE), Department of Biomedical Engineering, Universidad Politecnica de Catalunya, C/ Gran Capitan, s/n, 08034 Barcelona, Spain; ^4^Department of Imaging and Applied Physics, Curtin University, Perth, WA 6102, Australia

Numerical methods in biomedical research belong to a rapidly developing field which provides a state-of-the-art tool for biomedical research and applications. Reliable predictions will lead to patient-specific simulations in the next decade to improve the diagnosis and treatment of diseases. The main focus of this special issue is on the interface between numerical methods and biomedical applications for the human body. It is also interesting to have quantitative analysis from the molecular up to the whole organ level. The goal of this special issue is to bring together experts in related fields of computational biomedical research, for example, multiscale flow modeling, blood flow propagation, fluid-solid coupling, inverse problems in biomechanics, high performance computing of multiphysics discretization schemes, cardiovascular biomechanics, and porous media, to both foster engagement across areas of numerical methods and to identify novel applications to challenging biomedical modeling problems. The special issue has 12 papers and the details of these papers are as follows.

One paper presents the state-of-the-art parallelization technique taking advantage of the unique anatomical fiber architecture of skeletal muscles. The authors demonstrate the model's capability of simulating different aspects of nonisometric muscle contraction and the chemoelectromechanical behavior in complex skeletal muscles such as the tibialis anterior muscle.

The construction of artificial muscles is one of the most challenging developments in recent biomedical science. Another paper discusses the theoretical Hill-type muscle and stability model in determining the force-velocity relations of different animal species, which are based on the literature data from biological experiments. It showed that an antagonistic muscle actuator can help in stabilising a single inverted pendulum model in favour of a control approach using a linear torque generator.

One of the papers shows how the computational lower extremity model based on the inverse dynamic analysis and an optimization technique can be used to investigate different muscle activities and joint force patterns in knee osteoarthritis (OA) patients during walking. It provides insight into biomechanical changes in OA patients and evaluation of the postoperative functional outcomes of the OA treatments.

Another paper describes the use of an indentation test in determination of in vivo mechanical characteristics of human skin. It proposes a triphasic in vivo model of intact skin based on a general phenomenological thermohydromechanical and physicochemical (THMPC) approach of heterogeneous media.

One of the papers develops a computational model based on measurements from a hypoxic neonatal calf model of pulmonary hypertension (PH) to investigate the interplay between vascular and ventricular measures in the setting of progressive PH. Good agreement was observed between the 2D numerical model and the animal measurements. In the authors' simulated disease treatment, the model suggests that targeting proximal vascular remodeling, and in turn stiffness, may aid in recovery by further reducing RV afterload and power requirements.

Another paper proposes the analytical solutions for the mathematical model in describing the formation of liver zones via Adomian decomposition method with a system of nonlinear integropartial differential equations. This paper may shed light on the mathematical aspects of the formation of liver zones and explaining the distribution of two types of the liver cells.

One of the papers suggests creating a standardized nasal cavity model for adult Malaysian females. Computational fluid dynamic (CFD) analysis was performed to better understand the characteristics of the standardized model. The Malaysian female standardized model is larger in cross-sectional area when compared to the standardized Caucasian model, thus leading to lower average velocity magnitudes.

The accuracy of the numerical result is closely related to mesh density as well as its distribution. The aim of an another paper is to evaluate the hybrid mesh with unstructured mesh and study its effect on the flow parameters inside the nasal cavity by considering the complexity of its anatomical architecture. The hybrid mesh reported lower grid convergence index (GCI) than the unstructured mesh and thus its usefulness in nasal airflow studies.

The mathematical analysis for optimizing numerical performance based on different time integration schemes that pertain to both the fluid and solid accelerations is presented in one of the papers. The choice of time integration schemes has a significant influence on the stability of fluid-structure interaction coupling. This implies that in addition to material and its geometric properties, the choice of time integration schemes is critical in determining the stability of the numerical computation.

Another paper studies an integrated myocyte-Isac-Fb electromechanical model to investigate the effect of fibroblasts (Fbs) and stretch activated ion channel current (Isac) on cardiac electrical excitation conduction and mechanical contraction. The numerical results indicated that Fbs and Isac coupling caused diverse effects on action potential morphology during repolarization, depolarized the resting membrane potential of the human atrial myocyte, slowed down wave propagation, and decreased strains in fibrotic tissue.

Medical schools can benefit from a tool, system, or method that will help instructors train students and assess their tactile proficiency throughout their education. The robotic lumbar spine has the potential to satisfy these needs in palpatory diagnosis. One of the papers demonstrates the dynamic model and nonlinear control of a 15-degree-of-freedom, cable-actuated robotic lumbar spine (RLS) that enables the solution of positive and continuous cable tensions for cable-actuated robots. 

Another paper investigates the multiobjective optimization design of spinal pedicle screws using neural networks and genetic algorithm to achieve an ideal with high bending and pullout performances simultaneously. The optimal design has significantly higher fatigue life and comparable pullout strength as compared with commercial screws.

In this special issue, we have provided examples of recent progress in computational and mathematical methods in biomedicine, for the benefit of students, researchers, healthcare professionals, and teachers. 

